# Pancreaticoduodenectomy After Preoperative Biliary Drainage: A Single-Center Cohort Study of Piperacillin/Tazobactam Plus Fluconazole Prophylaxis and Infectious Outcomes

**DOI:** 10.7759/cureus.108378

**Published:** 2026-05-06

**Authors:** Luca Ottaviani, Giovanni Crotti, Andrea Celotti, Angelo Pan, Gian Luca Baiocchi

**Affiliations:** 1 General Surgery, ASST Cremona, Cremona, ITA; 2 Clinical and Experimental Sciences, University of Brescia, Brescia, ITA; 3 Infectious Disease, ASST Cremona, Cremona, ITA

**Keywords:** antibiotic prophylaxis, bile culture, pancreaticoduodenectomy, preoperative biliary drainage, surgical infection

## Abstract

Background

Pancreaticoduodenectomy (PD) is associated with high postoperative morbidity, particularly infectious complications. In patients undergoing preoperative biliary drainage (PBD), bile contamination is common and may lead to a mismatch between standard cephalosporin-based prophylaxis and biliary pathogens.

Methods

We conducted a retrospective single-center cohort study including 65 patients undergoing PD between December 2021 and May 2025. Of these, 42/65 (64.6%) underwent PBD. Patients received perioperative antibiotic prophylaxis with cefazolin or piperacillin/tazobactam plus fluconazole, according to institutional protocols. Intraoperative bile cultures were obtained in 44/65 patients, yielding 39 positive cultures with multiple isolates. Infectious complications and major morbidity (Clavien-Dindo > IIIA) were assessed. Multivariable logistic regression was performed to identify predictors of infectious complications, with subgroup analysis in drained patients.

Results

Positive bile cultures were observed in 38/42 (90.5%) drained patients compared with 1/23 (4.3%) non-drained patients (p<0.001). A total of 39 positive bile cultures yielded multiple isolates, including *Enterococcus* spp. in 24/39 (61.5%) cultures and *Candida* spp. in 10/39 (25.6%) cultures. Cefazolin did not provide coverage for at least one isolate in 30/39 (76.9%) cultures, compared with 13/39 (33.3%) for piperacillin/tazobactam plus fluconazole. In multivariable analysis restricted to drained patients, piperacillin/tazobactam plus fluconazole was independently associated with fewer infectious complications (odds ratio (OR): 0.13; 95% confidence interval (CI): 0.02-0.92; p=0.041).

Conclusions

In patients undergoing PD after PBD, bile contamination is frequent and characterized by enteric organisms and a relevant fungal component. Under these conditions, cefazolin prophylaxis shows a high rate of expected non-coverage, whereas piperacillin/tazobactam plus fluconazole provides broader microbiological coverage and is associated with reduced infectious complications. These findings support a tailored prophylactic strategy based on biliary colonization in PBD patients.

## Introduction

Pancreatic cancer and periampullary malignancies continue to pose a major clinical challenge due to the combination of late diagnosis, biological aggressiveness, and the need for complex multimodal care. For tumors of the pancreatic head, distal bile duct, ampulla, or duodenum, pancreaticoduodenectomy (PD) remains the cornerstone of potentially curative-intent treatment when resectability criteria are met. However, even in high-volume centers, PD carries a persistently high morbidity rate, and the postoperative course is often shaped by the interaction between technique-related complications and infection-related events. Among the perioperative variables that may influence infectious risk, preoperative biliary drainage (PBD) is particularly debated. PBD is frequently employed in obstructive jaundice to relieve cholestasis, treat cholangitis, facilitate neoadjuvant therapy, or optimize preoperative status. Yet, meta-analytic evidence has repeatedly suggested that PBD can increase postoperative complications in certain contexts [1,2], and more recent studies emphasize that the impact of PBD may be heterogeneous, potentially influenced by factors such as the clinical indication, the duration of drainage, neoadjuvant therapy, and the type of stent used [3]. A fundamental mechanistic concern is duodenobiliary reflux and bacterial ascent after drainage, leading to bile contamination; intraoperative spill of contaminated bile during PD has been proposed as a plausible driver of postoperative surgical site infection (SSI) and organ/space infections [4]. Antimicrobial prophylaxis is a key component of SSI prevention and is typically guided by procedure class and expected flora. Widely adopted guidance documents and SSI prevention frameworks generally endorse first- or second-generation cephalosporins for many clean-contaminated abdominal operations, coupled with correct timing and limited duration to support stewardship [5,6]. Yet PD, particularly when bile is colonized, may challenge the standard paradigm. In this setting, pathogens such as *Enterococcus* spp., Gram-negative rods, and resistant organisms are frequently reported, raising the possibility of a prophylaxis-pathogen mismatch. This mismatch has clinical relevance: discordance between perioperative prophylaxis and organisms later recovered from wound infections after PD has been described [7], implying that even technically sound prophylaxis delivery can be ineffective if the antimicrobial spectrum does not reflect the intraoperative contaminating flora. In parallel, literature has increasingly examined broader-spectrum prophylaxis strategies (e.g., piperacillin/tazobactam-based regimens) and their potential to reduce infectious outcomes after PD [8-13]. A final and often underappreciated issue is fungal colonization in bile, particularly *Candida* spp. While guidelines do not generally recommend routine antifungal prophylaxis for elective abdominal surgery, evidence suggests that in selected high-risk surgical populations, and depending on local epidemiology, fluconazole prophylaxis may reduce invasive candidiasis [14,15]. Recent surgical series have begun exploring antibiotic-antifungal strategies adapted to PBD status in pancreatic head resections [16,17]. Against this evolving background, we present a single-center analysis that links bile culture findings with prophylaxis regimens and clinical outcomes in PD, with a particular focus on patients undergoing PBD.

## Materials and methods

We conducted a retrospective, single-center analysis at the General Surgery Unit of ASST Cremona (Cremona, Italy), including all consecutive patients aged ≥18 years who underwent pancreaticoduodenectomy (open or laparoscopic) for any surgical indication between December 2021 and May 2025 (n=65). Patients in whom the procedure was planned but not performed after incision were excluded. Individuals were categorized according to preoperative biliary drainage status: 42 patients (64.6%) underwent PBD, either an endoscopic biliary stent and/or percutaneous transhepatic biliary drainage (PTBD) before surgery, whereas 23 patients (35.4%) proceeded to upfront surgery without any biliary drainage. Indications for preoperative biliary drainage included obstructive jaundice with cholangitis, severe hyperbilirubinemia requiring optimization before surgery, planned neoadjuvant therapy, delayed surgery, or referral after drainage performed at another institution. No formal enhanced recovery after surgery (ERAS) protocol change was introduced during the study period. Surgical indications, reconstruction strategy, perioperative infectious disease consultation, and postoperative monitoring followed the same institutional pathways. However, gradual improvements in multidisciplinary care and accumulated surgical experience over time cannot be excluded. Institutional prophylaxis was administered intravenously with the first dose delivered 30 minutes before skin incision, followed by intraoperative redosing every four hours, and discontinuation within 24 hours postoperatively. In cases of major blood loss (>1.5 L), additional dosing was provided to maintain effective serum and tissue concentrations throughout the procedure, consistent with pharmacologic principles that underpin the effectiveness of prophylaxis during prolonged operations. During 2021-2023, cefazolin (2 g) was predominantly used in both drained and non-drained patients, with individualized changes when allergies or concurrent antimicrobial therapies were present. Beginning in 2024, prophylaxis was adapted to drainage status: patients with PBD received piperacillin/tazobactam 4 g/0.5 g plus fluconazole 400 mg, while non-drained patients generally continued cefazolin-based prophylaxis. This change reflected the local recognition that PBD is strongly associated with contaminated bile and that bile isolates frequently include organisms for which cefazolin provides suboptimal or absent coverage. This protocol change was implemented as an institutional policy rather than left to individual surgeon preference, except in cases of allergy, concurrent antimicrobial therapy, or other specific clinical considerations. When feasible, bile was collected intraoperatively, typically after controlled bile duct section, and sent for microbiological culture. Sampling was pursued systematically in drained patients, except when technical constraints limited collection (as occurred in selected minimally invasive procedures), and it was performed selectively in non-drained patients. Empirical postoperative therapy was initiated according to clinical suspicion of infection and subsequently adjusted, when appropriate, according to intraoperative bile cultures, postoperative cultures, antimicrobial susceptibility testing, and infectious disease consultation.

The primary endpoint was the occurrence of any postoperative infectious complication within 30 days, defined as at least one of the following: superficial, deep, or organ/space SSI; intra-abdominal abscess/peritonitis; postoperative cholangitis; pneumonia; urinary tract infection; catheter-related bloodstream infection/bacteremia. Secondary outcomes included major morbidity, defined as Clavien-Dindo >IIIA, surgical reintervention, postoperative pancreatic fistula (POPF), length of stay, 30-day readmission, and 30-day mortality. POPF was graded according to the International Study Group of Pancreatic Surgery (ISGPS) 2016 update. Postoperative complications were classified according to the Clavien-Dindo classification system [18].

Statistical analysis

Categorical variables were compared using chi-square testing, while continuous variables were evaluated using Mann-Whitney U tests. Multivariable binomial logistic regression was performed both in the entire cohort and within the drained subgroup to identify predictors of infectious complications and major morbidity. Analyses were performed with Jamovi software version 2.6.22 (The Jamovi Project, Sydney, Australia).

## Results

A total of 65 patients undergoing pancreaticoduodenectomy between December 2021 and May 2025 were included and stratified according to PBD status. Overall, 42/65 patients (64.6%) underwent PBD (endoscopic stent and/or PTBD), whereas 23/65 (35.4%) proceeded directly to surgery without drainage. Within the drained cohort, endoscopic retrograde cholangiopancreatography (ERCP) stenting was the predominant modality (37/42, 88.1%), while PTBD was performed in 5/42 patients (11.9%). 

Preoperative characteristics are summarized in Table 1. The overall population was elderly, with a median age of 73 years (interquartile range (IQR), 64-79), with a non-significant trend toward higher age in drained patients (median 74.5 vs 70.0 years; p=0.07). Patients aged ≥70 years accounted for 61.5% of the total patients (40/65), including 28/42 (66.7%) in the drained group and 12/23 (52.2%) in the non-drained group (p=0.378). Among the patients, there were 38/65 women (58.5%), with a higher proportion in the non-drained group (17/23, 73.9%) compared with the drained patients (21/42, 50.0%), although this difference was not statistically significant (p=0.108).

**Table 1:** Preoperative characteristics of the entire population included in the study. Abbreviations: BMI, body mass index; ASA, American Society of Anesthesiologists; Preop RT, Preoperative radiotherapy; ERCP, endoscopic retrograde cholangiopancreatography; PTBD, percutaneous transhepatic biliary drainage; IQR, interquartile range. Statistical tests: Continuous variables were compared using the Mann-Whitney U test; categorical variables using the Chi-square or Fisher’s exact test, as appropriate.

Variable	Overall (n=65)	Drain (n=42)	No Drain (n=23)	Test	P-value
Age (years), median (IQR)	73 (64-79)	74.5 (64.2-80.0)	70.0 (63.5-74.5)	Mann-Whitney U	0.07
Age ≥70 years	40 (61.5%)	28 (66.7%)	12 (52.2%)	Chi-square	0.378
Male sex	27 (41.5%)	21 (50.0%)	6 (26.1%)	Chi-square	0.108
Female sex	38 (58.5%)	21 (50.0%)	17 (73.9%)	-	-
BMI (kg/m²), median (IQR)	23.0 (21.2-25.9)	23.4 (21.2-25.7)	22.4 (21.4-26.4)	Mann-Whitney U	0.47
BMI ≥25 kg/m²	16 (24.6%)	10 (23.8%)	6 (26.1%)	Fisher’s exact	1.000
ASA score ≥3	45 (69.2%)	28 (66.7%)	17 (73.9%)	Chi-square	0.746
Diabetes	14 (21.5%)	9 (21.4%)	5 (21.7%)	Fisher’s exact	1.000
Neoadjuvant therapy	23 (35.4%)	14 (33.3%)	9 (39.1%)	Chi-square	0.845
Preoperative radiotherapy	4 (6.2%)	3 (7.1%)	1 (4.3%)	Fisher’s exact	1.000
Jaundice at diagnosis	44 (67.7%)	42 (100.0%)	2 (8.7%)	Fisher’s exact	<0.001
ERCP	37 (56.9%)	37 (88.1%)	0 (0.0%)	Fisher’s exact	<0.001
PTBD	5 (7.7%)	5 (11.9%)	0 (0.0%)	Fisher’s exact	0.217
Previous abdominal surgery	36 (55.4%)	21 (50.0%)	15 (65.2%)	Chi-square	0.385
Modified Charlson index 3–4	19 (29.2%)	12 (28.6%)	7 (30.4%)	Fisher’s exact	1.000
Modified Charlson index ≥5	46 (70.8%)	30 (71.4%)	16 (69.6%)	Fisher’s exact	1.000

Median BMI was 23.0 kg/m² (IQR 21.2-25.9) and was comparable between groups (p=0.470); BMI ≥25 kg/m² was observed in 16/65 patients (24.6%) (10/42, 23.8% vs 6/23, 26.1%; p=1.000). The American Society of Anesthesiologists (ASA) score ≥3 was present in 45/65 patients (69.2%) (28/42, 66.7% vs 17/23, 73.9%; p=0.746), and diabetes in 14/65 (21.5%) (p=1.000). Neoadjuvant therapy was administered in 23/65 patients (35.4%) (14/42, 33.3% vs 9/23, 39.1%; p=0.845), while preoperative radiotherapy was reported in 4/65 (6.2%) patients (p=1.000).

Jaundice at diagnosis was observed in 44/65 patients (67.7%), almost exclusively in drained patients (42/42, 100.0% vs 2/23, 8.7%; p<0.001). Preoperative ERCP was performed in 37/65 patients (56.9%), all within the drained group (37/42, 88.1% vs 0/23; p<0.001), while PTBD was performed in 5/65 (7.7%), again only in drained patients (5/42, 11.9%; p=0.217).

Procedural characteristics and prophylaxis regimens are detailed in Table 2. Cefazolin-based prophylaxis was administered in 31/65 patients (47.7%), more frequently in non-drained patients (16/23, 69.6%) than in drained patients (15/42, 35.7%; p=0.019). Piperacillin/tazobactam (alone or in combination) was used in 26/65 patients (40.0%) (20/42, 47.6% vs 6/23, 26.1%; p=0.153), while piperacillin/tazobactam plus fluconazole was administered in 15/65 (23.1%), predominantly in drained patients (14/42, 33.3% vs 1/23, 4.3%; p=0.019). Other regimens were used in 8/65 patients (12.3%).

**Table 2:** Procedural factors. Bile culture = intraoperative bile sample collected for microbiological analysis. Statistical tests: Mann-Whitney U test (continuous variables); chi-square or Fisher’s exact test (categorical variables).

Variable	Overall (n=65)	Drain (n=42)	No Drain (n=23)	Test	P-value
Cefazolin prophylaxis	31 (47.7%)	15 (35.7%)	16 (69.6%)	Chi-square	0.019
Piperacillin/tazobactam (±combination)	26 (40.0%)	20 (47.6%)	6 (26.1%)	Chi-square	0.153
Piperacillin/tazobactam+fluconazole	15 (23.1%)	14 (33.3%)	1 (4.3%)	Fisher’s exact	0.019
Other regimens	8 (12.3%)	7 (16.7%)	1 (4.3%)	Fisher’s exact	0.152
Vascular resection	11 (16.9%)	7 (16.7%)	4 (17.4%)	Fisher’s exact	1.000
Operative time (min), median (IQR)	350 (321-397)	350 (320-385)	350 (329-407)	Mann-Whitney U	0.376
Laparoscopic approach	10 (15.4%)	3 (7.1%)	7 (30.4%)	Fisher’s exact	0.033
Open approach	55 (84.6%)	39 (92.9%)	16 (69.6%)	Fisher’s exact	0.033
Bile culture obtained	44 (67.7%)	39 (92.9%)	5 (21.7%)	Fisher’s exact	<0.001

Vascular resection was required in 11/65 patients (16.9%) (7/42, 16.7% vs 4/23, 17.4%; p=1.000). Median operative time was 350 minutes (IQR 321-397) and did not differ between groups (p=0.376). A laparoscopic approach was used in 10/65 patients (15.4%), more frequently in non-drained patients (7/23, 30.4% vs 3/42, 7.1%; p=0.033), while an open approach was performed in 55/65 patients (84.6%) (39/42, 92.9% vs 16/23, 69.6%; p=0.033).

Intraoperative bile cultures were obtained in 44/65 patients (67.7%), including 39/42 drained patients (92.9%) and 5/23 non-drained patients (21.7%) (p<0.001) (Table 2).

Overall, 39/65 patients (60.0%) had a positive bile culture. Among patients in whom bile cultures were obtained, 39/44 cultures (88.6%) were positive. Culture positivity was markedly higher in drained patients (38/42, 90.5%) compared with non-drained patients (1/23, 4.3%) (p<0.001). When restricted to sampled patients, 38/39 drained patients (97.4%) had positive cultures versus 1/5 non-drained patients (20.0%).

A total of 39 positive bile cultures were analyzed (Table 3), yielding multiple isolates per culture. *Enterococcus* spp. were identified in 24/39 cultures (61.5%), including *Enterococcus faecium* in 12/39 (30.8%) and *Enterococcus faecalis* in 5/39 (12.8%). *Escherichia coli* was identified in 15/39 cultures (38.5%). *Klebsiella pneumoniae* and *Klebsiella oxytoca* were each detected in 7/39 cultures (17.9%). *Candida* spp. were isolated in 10/39 cultures (25.6%), including *Candida albicans* in 6/39 (15.4%) and other species (e.g., *glabrata*, *tropicalis*, *krusei*, *parapsilosis*). *Pseudomonas aeruginosa* was identified in 4/39 cultures (10.3%). Less frequent isolates included *Serratia marcescens*, *Enterobacter cloacae* complex, *Hafnia alvei*, *Aeromonas* spp., and *Stenotrophomonas maltophilia*. Multiple microorganisms were frequently isolated within the same culture, consistent with polymicrobial colonization. Table 3 also summarizes expected antimicrobial non-coverage according to prophylactic regimen. Among the 39 positive bile cultures, cephalosporin-based prophylaxis did not cover at least one organism in 30/39 cultures (76.9%), whereas piperacillin/tazobactam plus fluconazole did not cover at least one organism in 13/39 cultures (33.3%). At the microorganism level, all *Enterococcus* spp. and *Candida* spp. isolates were not covered by cephalosporin-based prophylaxis, while residual non-coverage under piperacillin/tazobactam plus fluconazole remained limited to a minority of *Enterococcus* spp., *Candida* spp., and selected Gram-negative isolates.

**Table 3:** Expected antimicrobial non-coverage of microorganisms isolated from positive intraoperative bile cultures according to prophylactic regimen. n=39 positive bile cultures (not patients, not isolates). Numbers refer to the positive cultures containing at least one isolate of the specified organism. Multiple organisms could be isolated from the same culture (polymicrobial cultures).

Microorganism	Positive bile cultures containing organism (n=39)	Not covered by cephalosporin-based prophylaxis, n (%)	Not covered by piperacillin/tazobactam + fluconazole, n (%)
*Enterococcus* spp.	24	24 (100.0)	7 (29.2)
Escherichia coli	15	3 (20.0)	2 (13.3)
*Candida* spp.	10	10 (100.0)	1 (10.0)
Klebsiella oxytoca	7	0 (0.0)	1 (14.3)
Klebsiella pneumoniae	7	2 (28.6)	2 (28.6)
Pseudomonas aeruginosa	4	4 (100.0)	0 (0.0)

In Table 4, among the 39 positive bile cultures, cefazolin did not provide coverage for at least one isolate in 30/39 cultures (76.9%). In contrast, piperacillin/tazobactam plus fluconazole did not provide coverage for at least one isolate in 13/39 cultures (33.3%). 

**Table 4:** Expected antimicrobial coverage of bile isolates according to prophylactic regimens (culture-level analysis) Unit of analysis=positive bile cultures (n=39) Each culture was classified as “not covered” if at least one organism isolated from that culture was not expected to be covered by the prophylactic regimen, based on intrinsic spectrum of activity and available susceptibility results.

Outcome	No. of positive bile cultures (n=39)	Percentage (%)
≥1 organism not covered by cefazolin	30/39	76.9
≥1 organism not covered by piperacillin/tazobactam+fluconazole	13/39	33.3

Postoperative outcomes are summarized in Table 5. Major morbidity (Clavien-Dindo > IIIA) occurred in 17/65 patients (26.2%), including 10/42 (23.8%) in the drained group and 7/23 (30.4%) in the non-drained group (p=0.775).

**Table 5:** Postoperative complications and clinical outcomes. Statistical tests: Mann-Whitney U test (continuous variables); Chi-square or Fisher’s exact test (categorical variables).

Variable	Overall (n=65)	Drain (n=42)	No Drain (n=23)	Test	P-value
Clavien-Dindo > IIIA	17 (26.2%)	10 (23.8%)	7 (30.4%)	Chi-square	0.775
Postoperative pancreatic fistula	29 (44.6%)	18 (42.9%)	11 (47.8%)	Chi-square	0.901
Postoperative biliary fistula	10 (15.4%)	6 (14.3%)	4 (17.4%)	Fisher’s exact	1.000
Hemorrhage	14 (21.5%)	9 (21.4%)	5 (21.7%)	Fisher’s exact	1.000
Portal thrombosis	3 (4.6%)	2 (4.8%)	1 (4.3%)	Fisher’s exact	1.000
Delayed gastric emptying	10 (15.4%)	7 (16.7%)	3 (13.0%)	Fisher’s exact	0.978
Infectious complications	40 (61.5%)	25 (59.5%)	15 (65.2%)	Chi-square	0.854
Postoperative antibiotics	60 (92.3%)	38 (90.5%)	22 (95.7%)	Fisher’s exact	0.793
Length of stay (days), median (IQR)	23 (18–36)	23.5 (18–35.5)	22 (15–38)	Mann–Whitney U	0.636
Surgical reintervention	14 (21.5%)	7 (16.7%)	7 (30.4%)	Chi-square	0.329
30-day readmission	11 (16.9%)	6 (14.3%)	5 (21.7%)	Chi-square	0.674
30-day mortality	5 (7.7%)	3 (7.1%)	2 (8.7%)	Fisher’s exact	1.000

Postoperative pancreatic fistula occurred in 29/65 patients (44.6%) (18/42, 42.9% vs 11/23, 47.8%; p=0.901), while postoperative biliary fistula was observed in 10/65 (15.4%) patients (6/42, 14.3% vs 4/23, 17.4%; p=1.000). Hemorrhage occurred in 14/65 patients (21.5%) (9/42, 21.4% vs 5/23, 21.7%; p=1.000). Portal thrombosis was observed in 3/65 (4.6%) (2/42, 4.8% vs 1/23, 4.3%; p=1.000), and delayed gastric emptying in 10/65 (15.4%) (7/42, 16.7% vs 3/23, 13.0%; p=0.978).

Infectious complications occurred in 40/65 patients (61.5%), including 25/42 (59.5%) in drained patients and 15/23 (65.2%) in non-drained patients (p=0.854). Postoperative antibiotic therapy beyond prophylaxis was administered in 60/65 patients (92.3%) (38/42, 90.5% vs 22/23, 95.7%; p=0.793).

Median length of hospital stay was 23.0 days (IQR 18.0-36.0), with no significant difference between groups (23.5 vs 22 days; p=0.636). Surgical reintervention occurred in 14/65 patients (21.5%) (7/42, 16.7% vs 7/23, 30.4%; p=0.329). Thirty-day readmission occurred in 11/65 patients (16.9%) (6/42, 14.3% vs 5/23, 21.7%; p=0.674), and 30-day mortality in 5/65 patients (7.7%) (3/42, 7.1% vs 2/23, 8.7%; p=1.000).

Subgroup analysis restricted to drained patients is reported in Table 6. In multivariable logistic regression (n=42), prophylaxis with piperacillin/tazobactam plus fluconazole was independently associated with fewer infectious complications (OR: 0.133; 95% CI: 0.018-0.920; p=0.041), while no other variables were significantly associated with infectious outcomes.

**Table 6:** Multivariable logistic regression analysis for infectious complications in drained patients (n=42). RT: Radiotherapy; OR: Odds ratio; CI: confidence interval.

Variable	OR	95% CI	P-value
BMI >25	2.135	0.325-14.002	0.429
Female sex	0.959	0.188-4.901	0.96
Preop RT	1.779	0.105-30.120	0.69
Vascular resection	1.005	0.147-7.060	0.996
Surgical reintervention	4.094	0.310-54.113	0.285
Cefazolin prophylaxis	0.996	0.141-6.097	0.938
Piperacillin/tazobactam+fluconazole	0.133	0.018-0.920	0.041

## Discussion

This study provides a focused, clinically relevant contribution to the ongoing discussion on perioperative antibiotic prophylaxis in PD, particularly in patients undergoing PBD. Three main findings emerge and help frame the results in a pragmatic perioperative context. First, PBD was strongly associated with bile contamination. In this cohort, bile cultures were positive in nearly all drained patients, while contamination was rare in non-drained individuals. This finding reinforces the concept that biliary drainage facilitates duodenobiliary reflux and bacterial ascent, effectively converting the biliary tree into a reservoir of enteric microorganisms. Intraoperative exposure to contaminated bile during transection and reconstruction may therefore represent a relevant biological mechanism underlying postoperative surgical site and organ-space infections after PD [4]. Second, the microbiological profile observed in the drained patients provides a clear rationale for questioning standard cephalosporin-based prophylaxis in this setting. *Enterococcus* spp. predominated among bile isolates, and *Candida* spp. were detected in a substantial proportion of cases. As cefazolin lacks activity against enterococci and fungi, a high rate of prophylaxis-pathogen mismatch was expected and confirmed, with more than three-quarters of positive bile cultures containing at least one organism not covered by cephalosporins. In contrast, piperacillin/tazobactam plus fluconazole substantially reduced the proportion of predicted non-coverage, suggesting a closer alignment between prophylaxis spectrum and intraoperative contaminating flora in PBD patients. Third, this microbiological alignment translated into a clinically relevant signal. In the subgroup of drained patients, piperacillin/tazobactam plus fluconazole was independently associated with fewer postoperative infectious complications. This observation is consistent with an evolving body of evidence challenging cephalosporin-centered prophylaxis strategies in PD. Randomized data have demonstrated advantages of piperacillin/tazobactam over cephalosporin-based regimens [7], and observational studies have reported lower SSI rates when broader-spectrum antibiotics are used in jaundiced or stented patients [8]. Moreover, growing evidence links biliary pathogens not only to SSI but also to downstream complications such as postoperative pancreatic fistula, supporting a microbiologically driven model of postoperative morbidity after PD [11]. The fungal component warrants cautious interpretation. *Candida* spp. accounted for 25% of bile isolates in this series and contributed to the local decision, following infectious disease consultation, to include fluconazole in the prophylaxis regimen for drained patients from 2024 onward. While antifungal prophylaxis is not routinely recommended for elective abdominal surgery, selected high-risk surgical populations may benefit depending on local epidemiology [14,15]. PD-specific data remain limited, and previous series adapting antibiotic and antifungal strategies to PBD status have shown mixed results [16]. Accordingly, our findings should not be interpreted as support for universal antifungal prophylaxis, but rather as evidence in favor of a selective, epidemiology-driven approach in carefully defined high-risk subsets. Finally, the interaction between infection and technical complications remains central in PD. Emerging data suggest that early bacterial contamination, including *E. faecalis*, may contribute to fistula development and subsequent infection-related morbidity [17]. Within this framework, perioperative prophylaxis should be viewed as one component of a broader prevention strategy that also includes meticulous contamination control, standardized surgical technique, and prompt culture-guided escalation of therapy when infection occurs.

This study has some limitations. Its retrospective, single-center design and modest sample size naturally limit the strength of causal inference. In addition, a protocol adjustment introduced in 2024 may have contributed to temporal variability in practice patterns. Bile cultures were obtained far more frequently in drained than in non-drained patients, which may have led to underestimation of colonization in the latter group. Postoperative antibiotics were commonly used, potentially attenuating the isolated effect of perioperative prophylaxis on infectious outcomes. The apparently high overall infectious complication rate should be interpreted in light of the broad endpoint definition, which included both procedure-related infections and remote infections such as pneumonia and urinary tract infection.

Despite these considerations, the internal consistency between bile microbiology, antimicrobial coverage patterns, and clinical associations supports the biological plausibility of the findings and provides a reasonable basis for future prospective evaluation.

## Conclusions

In this single-center cohort, preoperative biliary drainage was strongly associated with bile colonization characterized by enteric organisms and a meaningful prevalence of *Candida* species. Under these microbiological conditions, cefazolin prophylaxis demonstrated a high rate of expected non-coverage, whereas piperacillin/tazobactam plus fluconazole provided broader predicted coverage and was independently associated with fewer infectious complications in drained patients. Given the study’s methodological constraints, including small sample size, temporal confounding, and high postoperative antibiotic use, these findings should be viewed as preliminary. These findings do not establish universal superiority of piperacillin/tazobactam plus fluconazole, nor do they support routine antifungal prophylaxis in all patients undergoing pancreaticoduodenectomy. Rather, they suggest that in patients with PBD, where local bile cultures show frequent *Enterococcus* spp. and *Candida* spp. colonization, institutional prophylaxis adapted to local microbiology may reduce prophylaxis-pathogen mismatch and may be associated with fewer infectious complications. Larger prospective studies are required.
